# CK2-mediated phosphorylation of Che-1/AATF is required for its pro-proliferative activity

**DOI:** 10.1186/s13046-021-02038-x

**Published:** 2021-07-15

**Authors:** Valeria Catena, Tiziana Bruno, Simona Iezzi, Silvia Matteoni, Annalisa Salis, Cristina Sorino, Gianluca Damonte, Maurizio Fanciulli

**Affiliations:** 1 SAFU Laboratory, Department of Research, Advanced Diagnostics and Technological Innovation, Translational Research Area, IRCCS Regina Elena National Cancer Institute, Via E. Chianesi 53, 00144 Rome, Italy; 2 Unit of Cellular Networks and Molecular Therapeutic Targets, Department of Research, Advanced Diagnostics and Technological Innovation, Translational Research Area, IRCCS Regina Elena National Cancer Institute, Via E. Chianesi 53, 00144 Rome, Italy; 3 Department of Experimental Medicine (DIMES), Biochemistry Section, University of Genoa, Viale Benedetto XV 1, 16132 Genoa, Italy

**Keywords:** Che-1, Cancer cells, Cell proliferation, Histone acetylation, Phosphorylation, CK2, SV40 LT

## Abstract

**Background:**

Che-1/AATF (Che-1) is an RNA polymerase II binding protein involved in several cellular processes, including proliferation, apoptosis and response to stress. We have recently demonstrated that Che-1 is able to promote cell proliferation by sustaining global histone acetylation in multiple myeloma (MM) cells where it interacts with histone proteins and competes with HDAC class I members for binding.

**Methods:**

Site-directed Mutagenesis was performed to generate a Che-1 mutant (Che-1 3S) lacking three serine residues (Ser^316^, Ser^320^ and Ser^321^) in 308–325 aa region. Western blot experiments were conducted to examine the effect of depletion or over-expression of Che-1 and Che-1 3S mutant on histone acetylation, in different human cancer cell lines. Proliferation assays were assessed to estimate the change in cells number when Che-1 was over-expressed or deleted. Immunoprecipitation assays were performed to evaluate Che-1/histone H3 interaction when Ser^316^, Ser^320^ and Ser^321^ were removed. The involvement of CK2 kinase in Che-1 phosphorylation at these residues was analysed by in vitro kinase, 2D gel electrophoresis assays and mass spectrometry analysis.

**Results:**

Here, we confirmed that Che-1 depletion reduces cell proliferation with a concomitant general histone deacetylation in several tumor cell lines. Furthermore, we provided evidence that CK2 protein kinase phosphorylates Che-1 at Ser^316^, Ser^320^ and Ser^321^ and that these modifications are required for Che-1/histone H3 binding. These results improve our understanding onto the mechanisms by which Che-1 regulates histone acetylation and cell proliferation.

**Conclusions:**

Che-1 phosphorylation at Ser^316^, Ser^320^ and Ser^321^ by CK2 promotes the interaction with histone H3 and represents an essential requirement for Che-1 pro-proliferative ability.

**Supplementary Information:**

The online version contains supplementary material available at 10.1186/s13046-021-02038-x.

## Background

Che-1/AATF (Che-1) is a highly conserved nuclear protein over-expressed in several cancerous tissues, such as prostate, lung, colon, lymphomas as well as in multiple myeloma (MM) and in hepatocellular carcinoma (HCC), where its levels increase during disease progression [1–5]. It has been previously demonstrated that Che-1 is involved in important cellular activities, including proliferation, regulation of cell cycle, apoptosis and stress response [6–11]. Many studies have highlighted the ability of Che-1 to affect the transcriptional machinery by its association with several proteins, such as RNA Polymerase II, the retinoblastoma protein (Rb), p65 and STAT3 [7, 12, 13]. Importantly, Che-1 contains an acidic region spanning from 308 to 325 aa highly homologous to HDAC proteins and several viral oncoproteins (SV40 Large T, E1A) by which it contacts Rb, thus inhibiting its binding to HDAC 1 and promoting cell proliferation [12, 14]. More recently, we have demonstrated that this region is required for displacing HDAC proteins from histones, increasing chromatin accessibility and MM cell proliferation [15]. Several post-translational modifications are able to modulate Che-1 half-life and functions [13, 16, 17], and the phosphorylation at specific serine or threonine residues has been associated with Che-1 activity in response to different stimuli, including induction of DNA damage or apoptosis [6, 18].

Casein kinase II (CK2) is a ubiquitous highly pleiotropic serine/threonine protein kinase present in cells in a tetrameric form consisting of two catalytic subunits (CK2α and its isoform CK2α’) and two regulatory β subunits [19]. It is expressed in all tissues of eukaryotic organisms and localized in different cell compartments [20]. Due to its ability to phosphorylate a wide variety of substrates, CK2 is involved in many cellular pathways, including cell growth, proliferation and survival [21–23]. Several studies highlighted that CK2 is up-regulated in different tumors, including kidney, head and neck, lung and prostate [24] and it is considered an attractive target to counteract drug resistance in cancer [19]. Indeed, a rising number of CK2 inhibitors have been developed and their effectiveness proven alone or in combination with conventional anticancer drugs supporting their possible application in clinical therapy [19]. Moreover, several studies have demonstrated that this kinase can interact with and regulate many factors involved in chromatin remodelling [25, 26].

In this study, we demonstrate that Che-1 promotes histone acetylation and cell proliferation not only in MM cells, and that the phosphorylation at three serine residues, Ser^316^, Ser^320^ and Ser^321^, is required for these activities. Notably, we identified CK2 kinase as the enzyme responsible for these modifications.

## Methods

### Cell culture and transfections

Human HCT116, HeLa, 293 T and U2OS cell lines were cultured in Dulbecco’s Modified Eagle’s Medium (DMEM, Euroclone) high glucose supplemented with 10% fetal bovine serum (FBS, Thermo Fisher Scientific), 2 mM glutamine (Thermo Fisher Scientific) and 40 μg/ml gentamicin. All cell lines were cultured at 37 °C, in a humidified atmosphere with 5% CO_2_. Mycoplasma contamination was periodically tested by RT-PCR analysis, using the following primers:

- Forward: 5′ - ACT CCT ACG GGA GGC AGC AGT A - 3′
- Reverse: 5′ - TCG ACC ATC TGT CAC TCT GTT AAC - 3′

Transfection experiments were carried out by using Lipofectamine 3000 Transfection System (Thermo Fisher Scientific) following the manufacturer’s instructions. Cells were analysed 48 h after transfection by western blot (WB) or immunofluorescence. Cell number and viability were assessed by Trypan Blue staining using Countess automated cell counter (Thermo Fisher Scientific).

### Recombinant plasmids and reagents

Myc-Che-1 has already been described [12, 27]. pCI-HDAC 1 was a kind gift from Dr. Sartorelli, while pSG5 Large T (pSG5 SV40 LT) plasmid was a gift from William Hahn (Addgene plasmid #9053; http://n2t.net/addgene:9053; RRID: Addgene_9053). Myc-Che-1 3S and pSG5 SV40 LT 3S were generated by in vitro mutagenesis using the QuikChange site-directed Mutagenesis system (Agilent Technologies) following the manufacturer’s instructions. PCR reactions were achieved using the following primers:

Myc- Che-1 3S:

- Forward 5′ - GCCCAATGCGGGAGGTGAGGAGATTGCTGGTGAAGATGATGAGC - 3′
- Reverse 5′ - GCTCATCATCTTCACCAGCAATCTCCTCACCTCCCGCATTGGGC - 3′

pSG5 SV40 LT 3S:

- Forward 5′ - AACCTGTTTTGCGCAGAAGAAATGCCAGCTGGTGATGATGAGGCT - 3′
- Reverse 5′ - AGCCTCATCATCACCAGCTGGCATTTCTTCTGCGCAAAACAGGTT - 3′

All mutations were confirmed by sequencing realized by Eurofins Genomics.

Stealth siRNA oligonucleotides targeting Che-1 (siChe-1), CSNK2A (siCK2), control sequence (siControl) and custom Che-1 3’UTR (sense 5′-CCCGCCUUUAAACGCCACAAAUAAA-3′; antisense 5′-UUUAUUUGUGGCGUUUAAAGGCGGG-3′) were purchased from Thermo Fisher Scientific. TBB (4,5,6,7 – Tetrabromobenzotriazole) was purchased from SelleckChem. Casein kinase II (CK2 - P60105) recombinant protein and Adenosine 5′-triphosphate (ATP - P07565) were purchased from New England BioLabs.

### Cell extracts, immunoprecipitation and RNA isolation

Total, cytoplasmic and nuclear extracts were prepared as previously described [13, 28, 29]. For immunoprecipitation (IP) experiments, nuclear extracts were resuspended in dilution buffer (50 mM TRIS pH 7.4, 150 mM NaCl, 5 mM EDTA, 10 mM NaF, 0.5% NP40, protease and phosphatase inhibitors). Dynabeads magnetic beads (Thermo Fisher Scientific) were incubated for 1 h at room temperature on a rotating wheel with the indicated antibodies. Nuclear extracts were then added to the beads and the incubation was continued for 2 additional hours. Immuno-complexes were purified by using a magnet (DynaMag-2, Thermo Fisher Scientific), the beads were washed five times with dilution buffer and eluted in 4X lithium dodecyl sulphate (LDS) sample buffer (Thermo Fisher Scientific) for WB analysis. Detailed information for all antibodies is provided in Supplementary Table S1. Total RNA was isolated from cells using EuroGOLD TriFast reagent (Euroclone) according to the manufacturer’s instructions. The amount of RNA was measured by using NanoDrop ND-1000 Spectrophotometer (Thermo Fisher Scientific).

### Western blot analysis

Samples were prepared as previously described [28], resolved on Bolt pre-cast 4-12% gels (Thermo Fisher Scientific), using Bolt MES SDS running buffer (Thermo Fisher Scientific) and transferred onto nitrocellulose membranes. After a blocking step in 5% non fat-dried milk in phosphate-buffered saline (PBS)-0.1% Tween, membranes were incubated with primary antibodies overnight at 4 °C. After three washes in PBS - 0.1% Tween, membranes were incubated with the appropriate HRP-linked secondary antibodies (Bio-Rad Laboratories) for 45 min at room temperature, washed with PBS-0.1% Tween and analysed by chemi-luminescence (GE Healthcare Life Science). Images were acquired and quantified using Alliance Mini HD6 system by UVITEC Ltd., Cambridge, equipped with UVI1D Software (UVITEC, 14-630,275). Detailed information for all antibodies is provided in the Supplementary Table S1.

### Immunofluorescence

Detection of both heterochromatin foci and chromatin modifications was performed as described in Bruno et al. [15]. Briefly, HCT116 cells were grown on cover slips, washed twice in PBS, fixed in 100% methanol for 5 min and then permeabilized for 1 h with 1% BSA, 10% normal goat serum, 0.3 M glycine in PBS - 0.1% Tween. Cells were incubated with anti-H3K9me3 overnight at 4 °C and stained 45 min with Alexa-Fluor-594-conjugated anti-rabbit secondary antibody (Thermo Fisher Scientific). For anti-Myc tag and anti-Che-1 immunofluorescences, cells were fixed in 4% formaldehyde for 10 min and then permeabilized with PBS - 0.2% Triton X-100 for 5 min at room temperature. Cells were stained for 2 h with the primary antibody, rinsed three times with PBS and stained respectively with Alexa-Fluor-594-conjugated anti-mouse or with Alexa-Fluor-488-conjugated anti-rabbit secondary antibodies (Thermo Fisher Scientific) 45 min at room temperature. Nuclei were visualized by staining with 1 μg/ml Hoechst dye 33258 (Sigma-Aldrich) for 10 min. 5-fluorouridine (5-FUrd) incorporation assay was performed as described in Sorino et al. [28]. Following incubation with 5 mM 5-FUrd (Sigma-Aldrich) for 10 min, cells were fixed in 2% formaldehyde for 10 min and then permeabilized with 0.5% Triton X-100 in PBS for 5 min at room temperature. Finally, after blocking in 3% bovine serum albumin (BSA) for 1 h, cells were stained with anti-BrdU antibody (Sigma-Aldrich). Images were acquired using a fluorescence microscope with a 40X objective (Zeiss, Germany) and processed with AxioVision 4.7.1 software.

### Flow cytometry

Cell apoptosis was detected using Annexin V-Fluorescein isothiocyanate (FITC)/propidium iodide (PI) double staining kit (Thermo Fisher Scientific) following the manufacturer’s instructions. HeLa cells were analysed 48 h post transfection with siControl or siChe-1 and the pellets were suspended in 200 μl Binding Buffer, and 5 μl Annexin V-FITC were added for 10 min at room temperature. After a wash, the cells were resuspended in 200 μl Binding Buffer and 10 μl PI were added for 5 min and analysed on a flow cytometer, MACSQuant Analyzer10 (Miltenyi Biotec) at an excitation wavelength of 488 nm.

### In vitro kinase assay

GST-Che-1 fusion protein was cloned into pGEX-4 T-3 vector as already described [13]. For in vitro kinase assay, GST-Che-1 was incubated with or without CK2 constitutively active for 1 h at 30 °C in slowly rotation on a rotating wheel in CK2 Reaction buffer (New England Biolabs) in presence of 200 μM ATP. Adding 4X LDS stopped reactions and the proteins were resolved by Bolt pre-cast 4-12% gels. The gel was stained with SimplyBlue SafeStain (Thermo Fisher Scientific) for 3 h at room temperature, and bands of interest were cut and then analysed by Nano - LC Mass spectrometry (MS).

### Nano - LC mass spectrometry

After kinase assay the bands of interest were cut from the gel, de-stained, reduced, alkylated and digested with trypsin following the procedure described by Shevchenko et al. [30]. The analysis of tryptic peptides was carried out by mean of nano-HPLC-MS/MS using an Ultimate 3000 nano-HPLC system and the obtained data were processed using an in-house software. The peptide pellets were re-suspended immediately before analysis and firstly loaded, from the sample loop, onto a trapping column (Acclaim PepMap C18; 2 cm × 100 μm × 5 μm, 100 Å- Thermo Fisher Scientific) using the loading eluents composition (95-5% ACN/H2O + 0.05% trifluoroacetic acid). The separation of peptides was performed at the flow rate of 300 nL/min and at a temperature of 35 °C using an Easy spray column (15 cm × 75 μm PepMap C18 3 μm, Thermo Fisher Scientific). A linear gradient of solution B (95-5% ACN/H2O + 0.08% formic acid) from 4 to 95% in 55 min was applied. All the analyses were performed in the positive ion mode. Single MS survey scans were performed in the Orbitrap, recording a mass window between 395 and 2000 m/z using a maximal ion injection time of 100 ms. The resolution was set to 70,000 and the automatic gain control was set to 3 × 10^6^ ions. The experiments were performed in data-dependent acquisition mode with alternating MS and MS/MS experiments. For MS/MS analysis an isolation window of 2 Da was used. CID was done with a target value of 5000 ions, a maximal ion injection time of 50 ms, normalized collision energy of 35%. Raw MS files were processed with the Thermo Scientific Proteome Discoverer software version 1.4. Peak list files were obtained by the SEQUEST search engine against the *Human protein database* containing both forward and reversed protein sequences. The resulting peptide hits were filtered for a maximum 1% FDR (false discovery rate) using the percolator tool. The database search parameters were: mass tolerance precursor 20 ppm, mass tolerance fragment CID 0.8 Da, dynamic modification of deamidation (N, Q), oxidation (M) and static modification of alkylation with IAM (C). Phosphorylation of serines was set as variable modifications. The software phosphoRS was used to validate the correct assignment of the phosphorylation sites. In any case, the option trypsin with two missed cleavages was selected [31].

### Identification by nano -HPLC -ESI-MS/MS

The data obtained by the HPLC separation and MS/MS analyses, carried out on digested samples, were submitted to the SEQUEST search engine against *Uniprot Human* protein database using the phosphoRS tool. This approach allowed the specific identification of *Tau-F of Apoptosis-antagonizing transcription factor (AATF/Che-1)*. Fifty-three high confidence peptides, corresponding to sequence coverage of 65.36% and high sequest score of 2391.39, were identified. From the MS/MS analysis it was possible to identify four phosphopeptides derived from Che-1, three of which were unambiguously assigned as phosphopeptides (Table 1). In a few cases it was not possible to assign the phosphate to specific serine residue(s), thus hindering the precise localization of the phospho-residue(s). The analysis of peptides obtained from Che-1 protein untreated allowed the identification fifty-four high confidence peptides corresponding to sequence coverage of 64.64% and high sequest score of 2082.81. As expected no phosphopeptides were found in this sample.

**Table 1:** CK2-phosphorylated Che-1 residues identified by nano-LC-mass spectrometry. MS/MS analysis of an in vitro phosphorylation assay showing Che-1 phosphopeptides including Ser^316^, Ser^320^ and Ser^321^. For each peptide the following details are reported: aminoacid sequence, modifications, phosphorylation site(s), pRS peptide score, pRS isoform probability, pRS site probability, *q*-value, PEP score and number of missed-cleavages. For details about the meaning of these parameters see Taus et al. [31]

Sequence	Modifications	Phospo-site	pRS Score	pRS Probability %	pRS Site Probabilities %	q-Value	PEP	# Missed Cleavages
TPGFSVQSIsDFEK	S10(Phospho)	S155	228	100.0	S (5): 0.0; S (8): 0.0; S (10): 100.0	0	1.8 × 10^− 5^	0
YLVDGTKPNAGSEEISsEDDELVEEK	S17(Phospho)	S320/S321	144	50.0	S (12): 0.0; S (16): 50.0; S (17): 50.0	0	4.3 × 10^− 5^	0
YLVDGTKPNAGsEEISSEDDELVEEKK	S12(Phospho)	S316	124	95.9	S (12): 95.9; S (16): 2.1; S (17): 2.1	0	9.3 × 10^− 4^	1
sLVGLqEELLFqYPDTR	S1(Phospho); Q6(Deamidated); Q12(Deamidated)	S288	95	100	S (1): 100.0	0	5 × 10^− 4^	0

### 2D gel electrophoresis

For 2D-Gel, cell pellets were resuspended in 2-D lysis buffer (2 M thiourea, 7 M urea, 4% CHAPS, 1% DTT, protease inhibitors cocktail) and sonicated. Total extracts were treated, where indicated, with Lambda Protein Phosphatase ( λ-PP) (New England BioLabs) following the protocol described in Yagamata et al., with minor modifications [32]. In short, cells pellets were re-suspended and 20 mM MnCl_2_ was added to 150 μg of protein extract in order to obtain a final concentration of 2 mM  λ-PP buffer and brought to a final volume of 20 μl with deionized water. The mixture was divided into two aliquots and 200 units of  λ-PP were added to one of the aliquots. After mixing, aliquots were incubated overnight at 30 °C under constant shaking and subsequently 100 μl of 2-D lysis buffer was added togheter with 1% sulfobetaine SB 4-7.

Isoelectric focusing was performed using 70 mm 4.0-7.0 linear Immobilized pH Gradient gel strips (IPG strips) (Bio-Rad Laboratories) on an electrophoresis unit (Ettan IPGphor 3; GE Healthcare Life Science). Equal amounts of proteins (70 μg) were loaded by passive in-gel rehydration for 12 h then run as follows: 1 h at 50 V, 30 min at 200 V, voltage gradient 30 min up to 1000 V, 20 min at 1000 V, voltage gradient 30 min up to 5000 V and 1 h at 5000 V. Before performing the 2nd electrophoresis dimension, IPG strips were equilibrated for 15 min at room temperature in 1% DTT to reduce the proteins, and sulfhydryl groups were subsequently derivatized using 4% iodoacetamide (both solutions were prepared in 50 mM Tris, pH 8.8, 6 M urea, 30% glycerol, 2% SDS, and 2% bromophenol blue) for 15 min. Strips were transferred to 1.0-mm-thick 4-12% pre-casted polyacrylamide mini-gels (Thermo Fisher Scientific) for the 2nd electrophoresis dimension and then gels were run at 130 V for 2 h. 2D gels were then transferred onto PVDF filters.

### In vitro GST pull down assay

Full length H3 open reading frame was cloned into EcoRI/XhoI sites of pGEX-4 T-1 in order to generate GST-H3 fusion protein. GST-H3 or GST control proteins were prepared following standard procedures and used for in vitro binding assay. Briefly, GST proteins were pre-incubated with Che-1 308-325, Che-1 3S or control peptides (Biomatik Corporation) for 1 h at 4 °C on a rotating wheel in interaction buffer (20 mM Tris pH 7.9, 300 mM NaCl, 0.2 mM EDTA, 0.1% NP40, 10 μg/ml BSA). Total extracts from HCT116 cells over-expressing pCI-HDAC 1 were then added to the GST/peptides complexes and the incubation was continued for three additional hours. Samples were eluted in 4X LDS sample buffer by heating for 10 min at 70 °C and then subjected to WB analysis. Peptides sequence commercially synthesized are:

- Control: LKGAKPIRPVVVKAPPA
- Che-1 308-325: DGTKPNAGSEEISSEDDEL
- Che-1 3S: DGTKPNAGAEEIAAEDDEL

### Micrococcal nuclease assay

Micrococcal nuclease (MNase) assay was performed using the protocol described in Zaret et al. [33]. Briefly, HCT116 cells were seeded in 100 mm plate and transiently transfected with Myc-Che-1 wt, Myc-Che-1 3S or empty vector. Twenty-four h post transfection cells were subjected to the protocol described in Bruno et al. [15].

### Quantification and statistical analysis

Data are presented as the mean of three independent experiments ± standard deviation (SD). Statistical analyses were performed using R software. Two-tailed Student’s *t*-tests with Benjamini-Hochberg correction were performed to compare one parameter between two groups. For the multiple group comparison, one-way ANOVA with Tukey HSD test was used. *P* < 0.05 was considered significant.

Statistical significance is indicated by asterisks as follows: *P < 0.05, **P < 0.01, ***P < 0.005, ****P < 0.001, n.s. = not significant.

## Results

### Che-1 depletion reduces cell proliferation

**Fig. 1:**
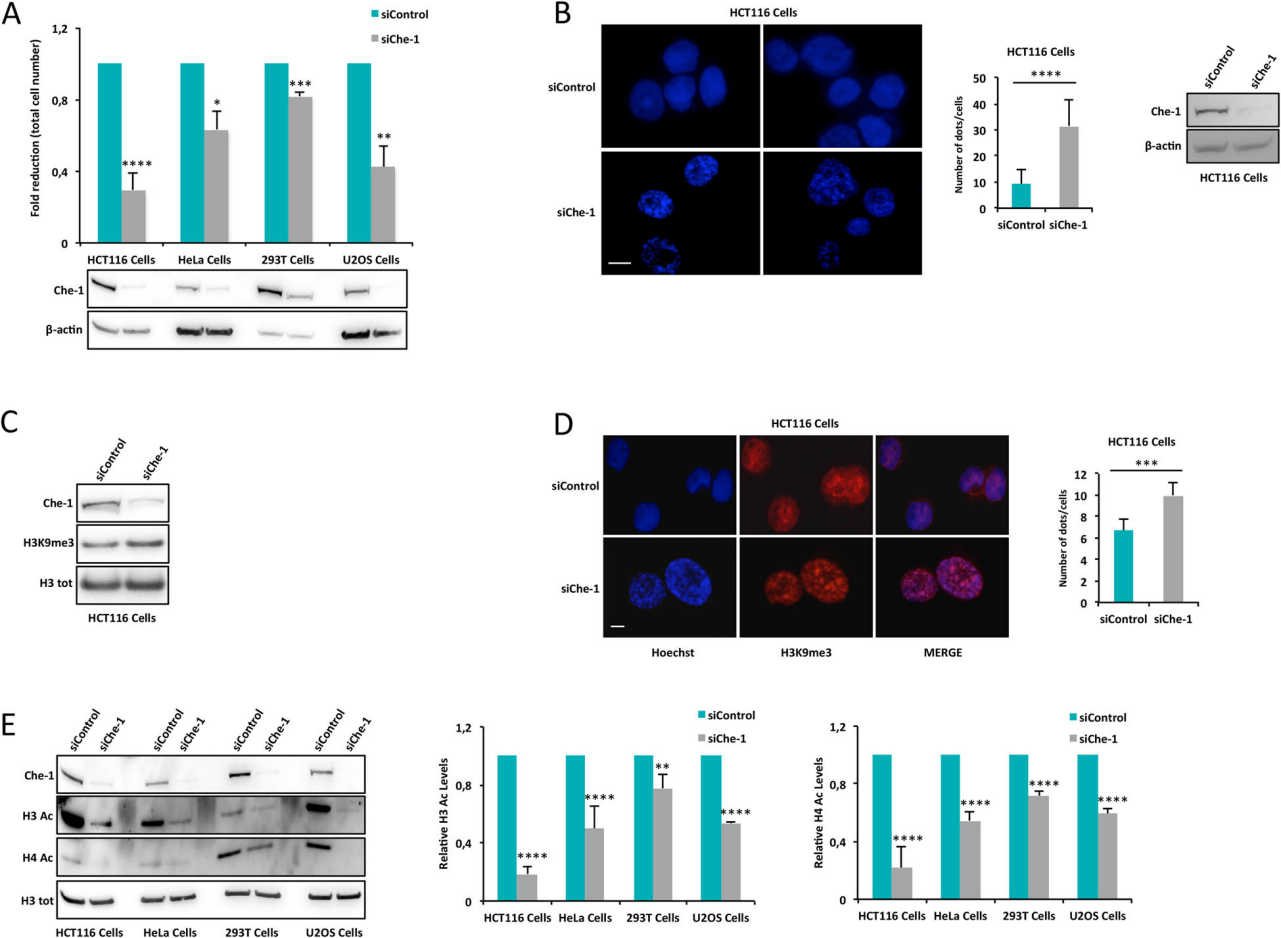
Che-1 depletion reduces cell proliferation. **A** Cell proliferation analysis (top) and WB analysis of total cell extracts (bottom) from the indicated cell lines transiently transfected with siRNA Control (siControl) or siRNA Che-1 (siChe-1) for 48 h. Bar plot shows the average of number of cells observed in these experiments (*n* = 3). **B** Two images representing nuclear morphology (left), corresponding fluorescence intensity analysis (middle) and WB analysis (right) of HCT116 cells transiently transfected with siControl or siChe-1. Nuclei were visualized by staining with Hoechst dye. Scale bar, 10 μm. Error bars represent the SD after combining the results of three different experiments. **C** Representative WB analysis with the indicated antibodies of nuclear extracts from HCT116 cells transfected as in B. **D** Representative immunofluorescence analysis (left) and corresponding fluorescence intensity analysis (right) of H3K9me3 expression in HCT116 cells transfected as in B. Nuclei were visualized by staining with Hoechst dye. Scale bar, 10 μm. **E** Representative WB analysis with the indicated antibodies of total cell extracts from the indicated cell lines transfected as in A (left). Relative H3 and H4 acetylated protein levels were calculated from three different experiments by densitometry (right). Statistical significance is indicated by asterisks as follow: *P < 0.05, **P < 0.01, ***P < 0.005, ****P < 0.001, n.s. = not significant

As previously observed, Che-1 displayed to have a key role in cell proliferation in solid and haematological tumors [5, 15, 34, 35]. To further characterize its proliferative effect, we performed cell proliferation analysis in cells of different origin, namely HCT116, HeLa, 293 T and U2OS cells, depleted or not for Che-1 expression. Because Che-1 depletion induces apoptosis [2, 15], all analyses were performed 48 h after small interfering RNA (siRNA) Che-1 transfection, when the apoptotic process was not yet activated (Supplementary Figures 1A-C) [15]. As shown in Fig. 1A, Che-1 inhibition produced a significant reduction of cell proliferation when compared to control cells in all tested cell lines. Since Che-1 depletion induces heterochromatin accumulation in MM cells [15], we evaluated whether this was a general phenomenon. To this aim, we analysed the formation of heterochromatin foci by immunofluorescence in HCT116 cells, observing that Che-1 depleted cells exhibited a strong increase of Hoechst-positive heterochromatin foci (from 9 to 32 foci/cell) (Fig. 1B). Consistent with these findings, WB analysis showed that Che-1 knockdown produced a significant increase of H3K9me3 levels, an important hallmark of heterochromatin (Fig. 1C). These results were confirmed through immunofluorescence experiments in which a marked increase of H3K9me3 positive dots was observed following Che-1 downregulation (Fig. 1D). It has been shown that Che-1 depletion produces a global reduction of histone acetylation in MM cells [15]. To extend these findings, we performed WB analyses in several cell lines depleted or not for Che-1 expression, observing that the loss of Che-1 expression produced a strong reduction in both H3 and H4 histone acetylation (Fig. 1E and Supplementary Figure 1D). In line with the obtained results, we observed a reduction of about 50% of total RNA in Che-1 depleted cells when compared to control cells (Supplementary Figure 1E). Overall, these findings reinforce the role of Che-1 in sustaining cell proliferation by maintaining an active state of chromatin.

### Che-1 is highly phosphorylated

**Fig. 2:**
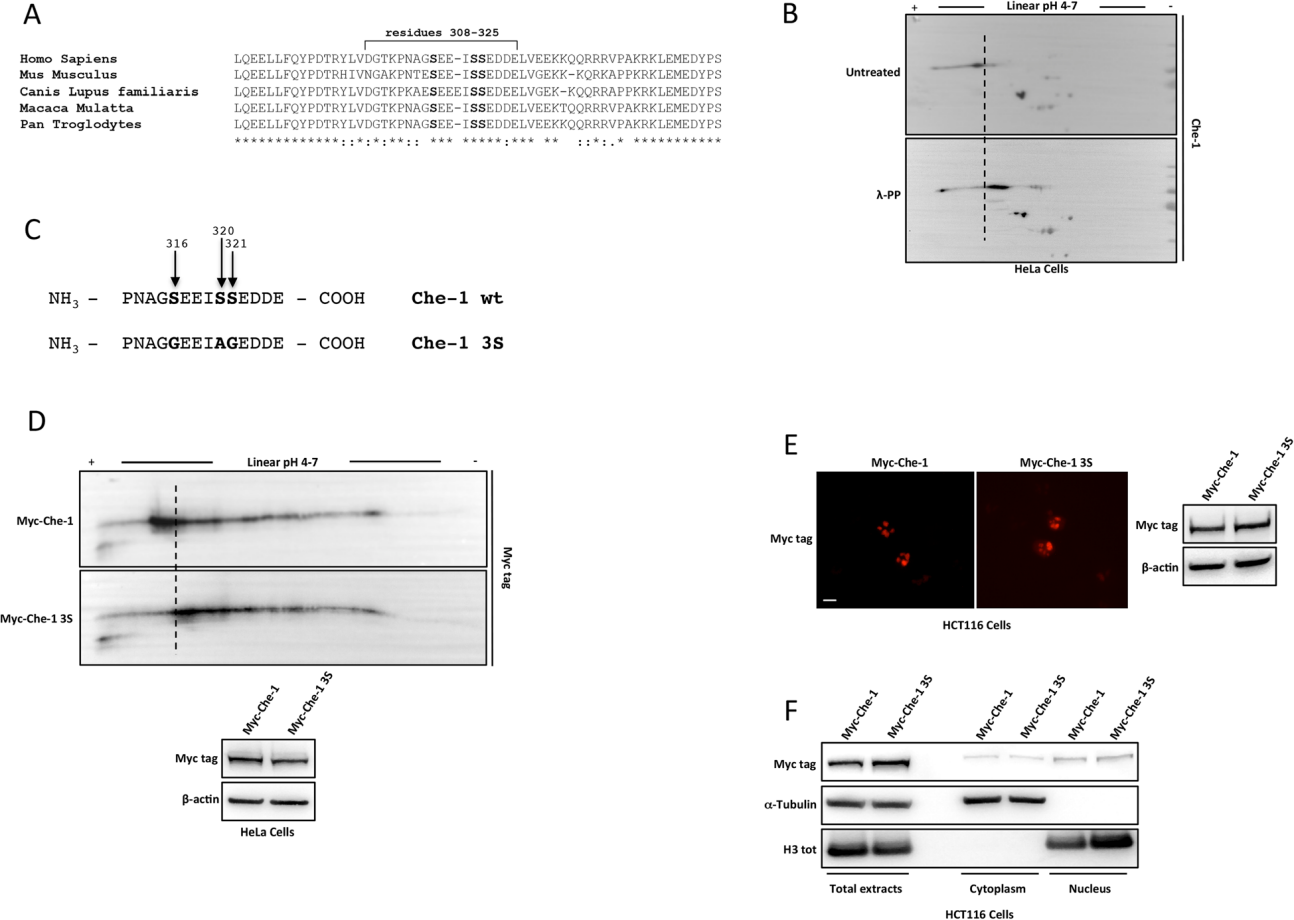
Che-1 is highly phosphorylated. **A** A multiple sequence alignment of five mammalian Che-1 proteins. Below each position of the protein sequence alignment is present a key denoting conserved sites (*), sites with conservative replacements (:), sites with semi-conservative replacements (.) and sites with non-conservative replacements (). **B** Total cell extracts from HeLa cells treated or not with λ-PP (see Methods) were separated onto parallel 2D-Gel electrophoresis. **C** Schematic representation of Che-1 point mutations (see Methods) at the indicated sites. **D** 2D-Gel electrophoresis (top) and representative WB (bottom) of HeLa cells transiently transfected with Myc-Che-1 or Myc-Che-1 3S. **E** Representative immunofluorescence images (left) and WB analysis (right) of HCT116 cells transfected with the indicated expression vectors. Scale bar, 10 μm. **F** WB analysis with the indicated antibodies of total, cytoplasmic and nuclear fractions from HCT116 cells transfected as in D

Che-1 affects multiple cellular processes thanks to its ability to interact with several transcription factors [7, 12]. These interactions are modulated by post-translational modifications in response to different stimuli, including phosphorylation at specific serine or threonine residues [6]. In this context, we have previously shown that Che-1 contains a highly acidic sequence (308-325 aa) involved in Che-1-histone binding. Indeed, Che-1 mutant lacking this region was no longer able to bind histone H3 [15]. Interestingly, in this region are present three highly conserved serine residues (in bold; Fig. 2A), whose phosphorylation has been reported by many phospho-proteomic studies [36–38]. To investigate the relevance of this Che-1 region, we performed a 2D gel electrophoresis analysis of total extracts from HeLa cells treated or not with lambda phosphatase (λ-PP), and observed that Che-1 is constitutively and highly phosphorylated (Fig. 2B and Supplementary Figure 2A). Next, we produced a Che-1 mutant (Che-1 3S) in which the serine residues in position 316, 320 and 321 were replaced with glycine or alanine (Fig. 2C). A bidimensional gel electrophoresis analysis of HeLa cells over-expressing Che-1 wild type (Myc-Che-1) or 3S mutant (Myc-Che-1 3S) showed that this mutant was less phosphorylated than wild-type (wt) protein (Fig. 2D and Supplementary Figure 2B), indicating that these residues contribute significantly to total protein phosphorylation.

Next, we started to characterize the functional role of these modifications investigating whether the 3S mutant had a different cellular localization. Immunofluorescence experiments showed that the mutant retained the peculiar nuclear signal of the wt protein (Fig. 2E). In agreement with these data, a subcellular fractionation of HCT116 cells transiently transfected with Myc-Che-1 wt or 3S mutant, detected a strong accumulation in the nuclear fraction of both molecules (Fig. 2F).

### Che-1 phosphorylation is required for its pro-proliferative ability

**Fig. 3:**
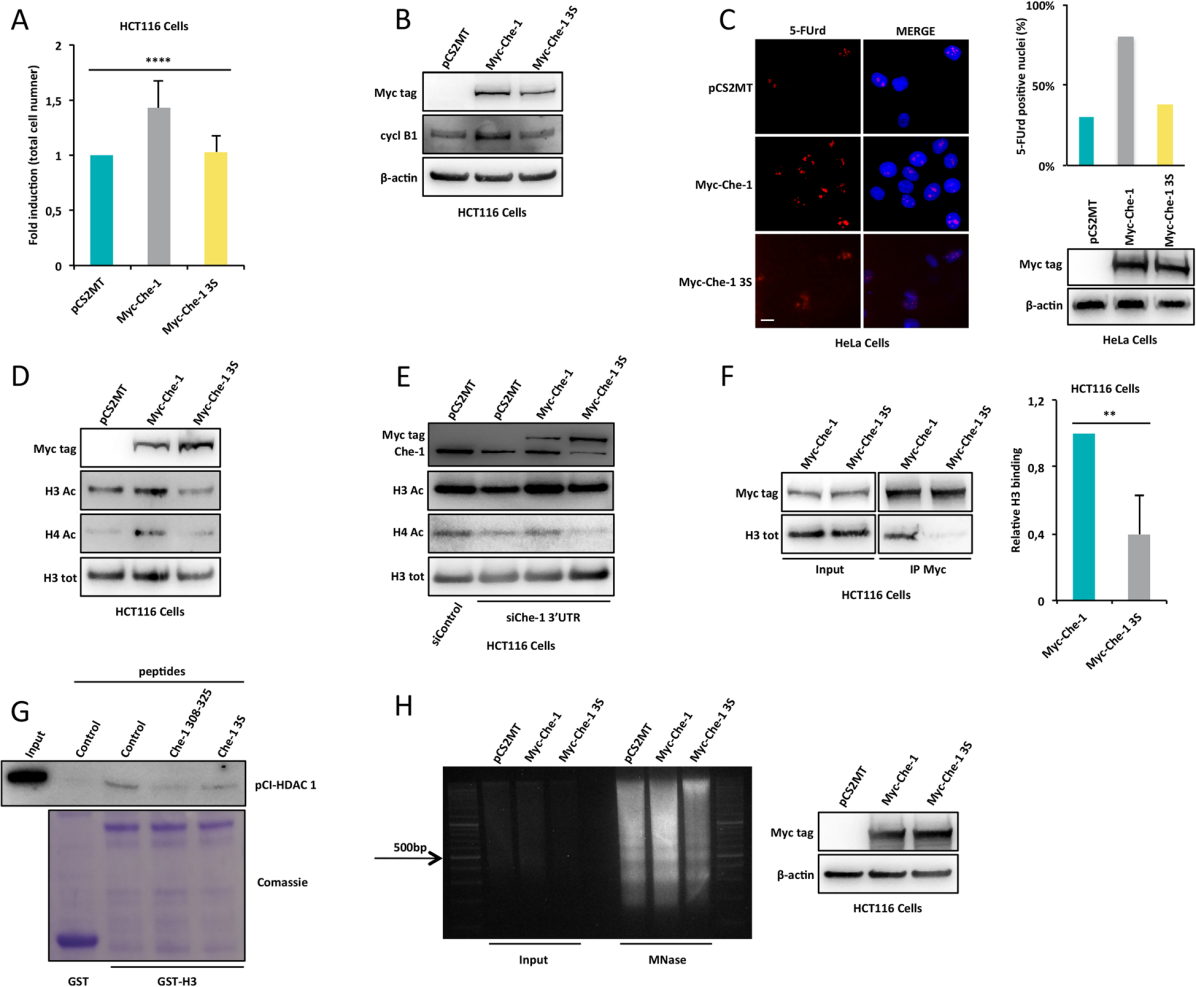
Che-1 phosphorylation is required for its pro-proliferative ability. **A** Cell proliferation analysis of HCT116 cells transiently transfected with Myc-Che-1, Myc-Che-1 3S or control vector (pCS2MT). Bar plot shows the average number of cells observed in these experiments (*n* = 6). **B** Representative WB analysis with the indicated antibodies of total cell extracts from HCT116 cells transfected as in A. **C** 5-FUrd labelled RNA was evaluated by immunostaining with anti-BrdU antibody in HeLa cells transiently transfected with the indicated expression vectors. Nuclei were stained with Hoechst dye. Scal bar, 10 μm (left). Bar plot showing the percentage of 5-FUrd positive nuclei (right, top). Percentage was calculated from 100 randomly selected cells for each group. Representative WB showing the transfection efficiency of the 5-FUrd incorporation assay described above (right, bottom). **D** WB analysis with the indicated antibodies of total cell extracts from HCT116 cells transfected as in A. **E** WB analysis of total cell extracts of HCT116 cells transfected with siControl or siChe-1 3’UTR alone or in combination with empty vector or with Che-1 expressing vectors (Myc-Che-1 and Myc-Che-1 3S). **F** Nuclear extracts from HCT116 cells transiently transfected with Myc-Che-1 wt or 3S mutant, were subjected to IP with anti-Myc monoclonal antibody. Immunoprecipitated complexes were then analysed by WB with the indicated antibodies. Input corresponds to 10% of the nuclear extracts used for IP (left). Relative H3 binding was calculated from three different experiments by densitometry. H3 intensity was normalized to the one of Myc-Che-1 wt or 3S mutant (right). **G** WB analysis of GST-pull down experiment conducted using total extracts from HCT116 cells over-expressing pCI-HDAC 1 incubated with purified GST-H3 fusion protein or control GST agarose beads, in presence or absence of the indicated peptides. GST fusion proteins expression is shown by Comassie blue staining. **H** MNase digestion pattern of nuclei (left) and corresponding WB analysis (right) with the indicated antibodies of total cell extracts from HCT116 cells transfected as in A. Statistical significance is indicated by asterisks as follow: *P < 0.05, **P < 0.01, ***P < 0.005, ****P < 0.001, n.s. = not significant

The data shown above demonstrate that three serine residues contained in the 308-325 region are phosphorylated. This region plays an important role in Che-1’s functions [12, 15]. To shed light on the relevance of these phosphorylations, we evaluated the effect of the over-expression of Myc-Che-1 wt or 3S mutant. As shown in Fig. 3A and Supplementary Figure 3A, Myc-Che-1 3S over-expression in HCT116 cells did not induce cell proliferation when compared to Myc-Che-1 wt. These results were confirmed in MM cells (KMS27 cells) and in other cell lines (Supplementary Figures 3B-D). The relevance of these three serine residues in the pro-proliferative role of Che-1 was further confirmed by evaluating the effect of the over-expression of these molecules on the levels of cyclin B1. In fact, as shown in Fig. 3B, only Myc-Che-1 wt was able to increase the levels of this protein. Next, we verified whether the over-expression of Myc-Che-1 3S had any impact on global transcription by measuring the level of total RNA. As shown in Supplementary Figure 3E, the Myc-Che-1 3S mutant was completely unable to activate transcription. These findings were further confirmed by in vivo pulse labelling with the nucleoside analogue 5-FUrd and by the analysis of its incorporation into nascent RNA through immunostaining. Indeed, as shown in Fig. 3C, cells over-expressing Myc-Che-1 wt exhibited a significant increase of 5-FUrd signal, which was not observed in cells transfected with Myc-Che-1 3S. In order to evaluate whether Che-1 phosphorylation in Ser^316^, Ser^320^ and Ser^321^ was required to sustain histone acetylation, we analysed the effect of Myc-Che-1 wt or 3S mutant over-expression on H3 and H4 histone acetylation. As shown in Fig. 3D, Myc-Che-1 3S mutant showed no effect on histone acetylation and remarkably it was unable to rescue the effects of endogenous Che-1 depletion (Fig. 3E). These results prompted us to investigate the ability of the Che-1 3S mutant to interact with histone H3. To this aim, we performed co-immunoprecipitation experiments with anti-Myc antibody using nuclear extracts from HCT116 cells over-expressing Myc-Che-1 wt or its 3S mutant. As shown in Fig. 3F and in Supplementary Figure 3F, the replacement of the three serine residues prevented Che-1/histone H3 interaction. Moreover, an in vitro competition assay demonstrated that only the Che-1 308-325 peptide containing phosphorylated serine residues was able to displace HDAC 1 from histone H3 (Fig. 3G). Notably, KMS27 cells transfected with Che-1 308-325, but not with Che-1 3S peptide, displayed a reduction in cell proliferation (Supplementary Figure 3G). Consistent with these results, an MNase susceptibility assay performed in HCT116 cells transiently transfected with Myc-Che-1 wt or 3S mutant demonstrated that the mutant over-expression produced a more compacted chromatin structure (Fig. 3H).

Overall, these results clearly indicate that the presence of the three serine residues in 308-325 aa region represents an essential requirement for Che-1 activity, promoting the Che-1/histone binding and histone acetylation.

### Che-1 and SV40 large T antigen display similar functions

**Fig. 4:**
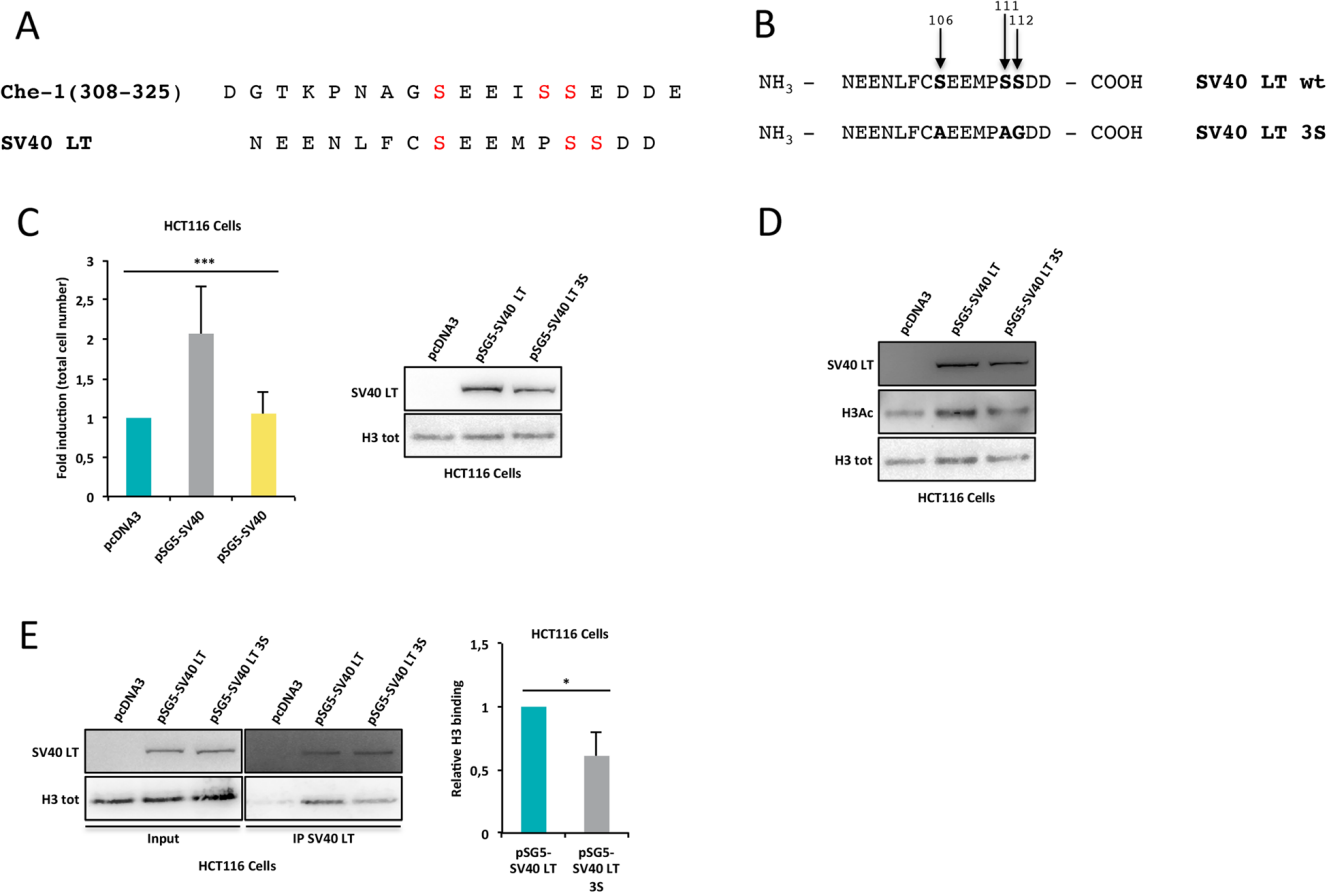
Che-1 and SV40 Large T antigen display similar functions. **A** Sequence alignment of Che-1 and SV40 Large T antigen (SV40 LT) proteins. Conserved serine residues are in red. **B** Schematic representation of SV40 LT point mutations (see Methods) at the indicated sites. **C** Cell proliferation analysis (left) and relative representative WB analysis (right) of HCT116 cells transfected with pSG5-SV40 LT wt, 3S mutant or empty vector (pcDNA3). Bar plot shows the average number of cells observed in these experiments (*n* = 3). **D** WB analysis with the indicated antibodies of total cell extracts from HCT116 cells transfected as in C. **E** Nuclear extracts from HCT116 cells transfected as in C and subjected to IP with SV40 antibody. Immunoprecipitated complexes were then analysed by WB with the indicated antibodies. Input corresponds to 10% of the nuclear extracts used for IP (left). Relative H3 binding was calculated from three different experiments by densitometry. H3 intensity was normalized to the one of SV40 wt or 3S mutant (right). Statistical significance is indicated by asterisks as follow: *P < 0.05, **P < 0.01, ***P < 0.005, ****P < 0.001, n.s. = not significant

SV40 LT is a multifunctional viral oncoprotein implicated in a wide range of cellular processes including transcriptional activation and repression, stimulation of the cell cycle and cell transformation [39]. Interestingly, SV40 LT expression increases global histone acetylation and CREB-binding protein (CBP) histone acetyltransferase (HAT) activity [39]. As already described, Che-1 308-325 aa region is highly conserved among mammalian species and shared with several viral and HDAC 1-3 proteins [12]. Among viral oncoproteins, SV40 Large T antigen (SV40 LT) contains three particular serine residues, in position 106, 110 and 111, corresponding to those of Che-1 (in red; Fig. 4A). These observations prompted us to verify whether SV40 LT phosphorylation on Ser^106^, Ser^110^ and Ser^111^ residues, was required for its ability to regulate cellular proliferation. To this aim, we produced a SV40 LT mutant (SV40 LT 3S) in which Ser^106^, Ser^110^ and Ser^111^ residues were replaced with alanine or glycine (Fig. 4B). Notably, HCT116 cells over-expressing SV40 LT 3S mutant failed to induce cell proliferation observed in SV40 LT wt cells (Fig. 4C). Furthermore, this mutant was unable to induce the histone acetylation increase observed in cells transfected with SV40 LT wt (Fig. 4D). Since Che-1 phosphorylation in the 308–325 region is required for the interaction with histone H3 (Fig. 3F), we evaluated whether SV40 LT wt was also able to interact with histone H3 and whether phosphorylation of Ser^106^, Ser^110^ and Ser^111^ residues was involved in this binding. To this aim, we performed co-immunoprecipitation experiments with anti-SV40 antibody in nuclear extracts from HCT116 transiently transfected with SV40 LT wt or its 3S mutant. Remarkably, we observed that the SV40 LT wt protein showed the ability to bind histone H3 whereas, in line with results obtained with Che-1, the mutant lost this ability (Fig. 4E).

Taken together, these data reinforce the importance of this highly acidic region in several proteins for binding to histones and cell proliferation.

### CK2 phosphorylates Che-1

**Fig. 5:**
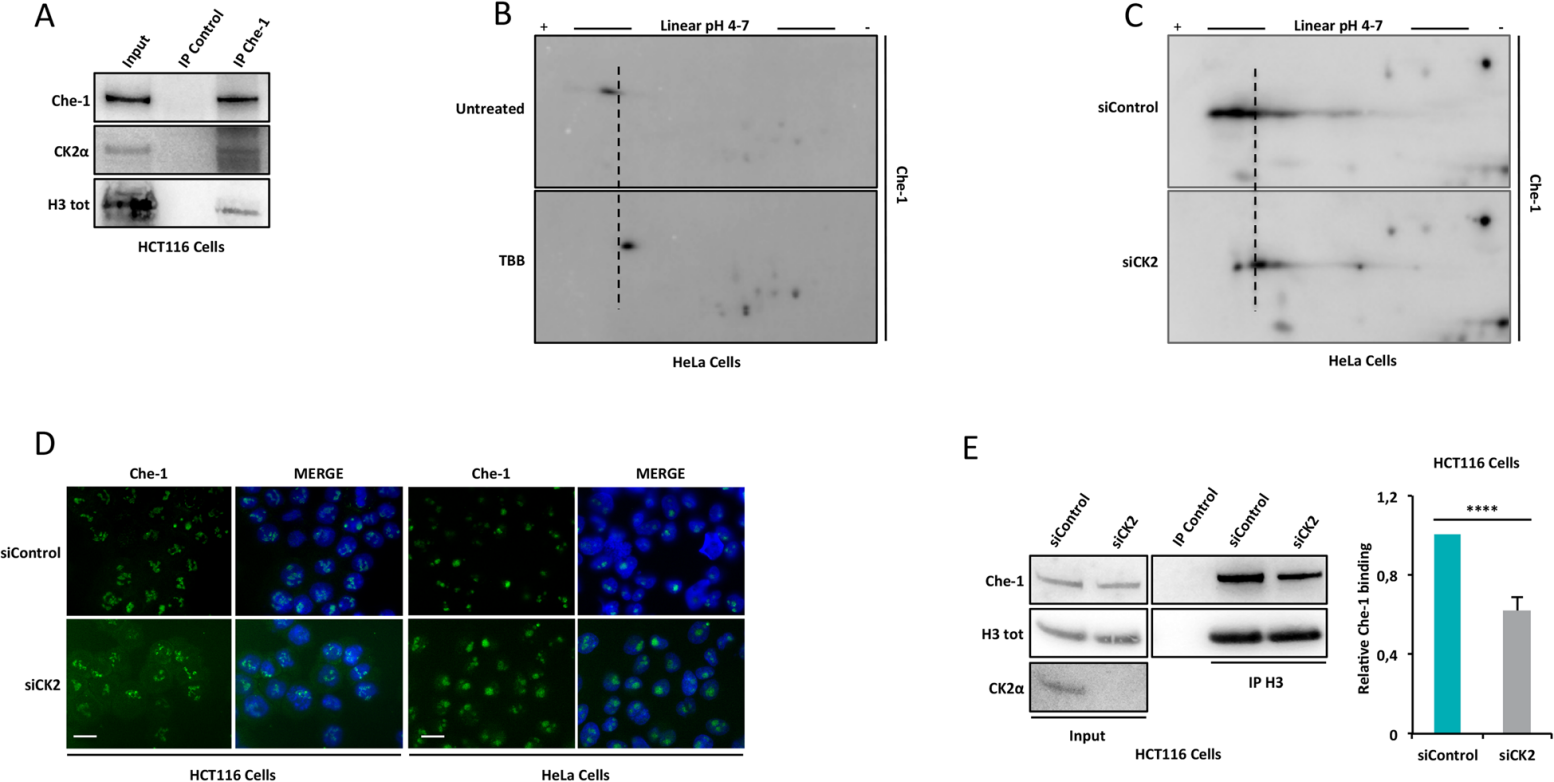
CK2 phosphorylates Che-1. **A** Nuclear extracts from HCT116 cells subjected to IP with Che-1 antibody. Immunoprecipitated complexes were then analysed by WB with the indicated antibodies. Input corresponds to 10% of the nuclear extracts used for IP. **B** and **C** 2D-Gel electrophoresis of total cell extracts from HeLa cells treated or not with 80 μM TBB for 4 h (B) or transfected with siControl or siCK2 (C). **D** Representative immunofluorescence analysis of Che-1 expression in the indicated cell lines transiently transfected with siControl or siCK2. Scale bar, 10 μm. **E** Nuclear extracts from HCT116 cells transiently transfected as in D and subjected to IP with H3 antibody. Immunoprecipitated complexes were then analysed by WB with the indicated antibodies (left). Input corresponds to 10% of the nuclear extracts used for IP. Relative Che-1 binding was calculated from three different experiments by densitometry. Che-1 intensity was normalized to the one of H3 (right). Statistical significance is indicated by asterisks as follow: *P < 0.05, **P < 0.01, ***P < 0.005, ****P < 0.001, n.s. = not significant

Next, we focused on identifying the kinase responsible for the phosphorylation of Che-1 in Ser^316^, Ser^320^ and Ser^321^ residues. Previous studies have demonstrated that protein kinase Casein Kinase II (CK2) phosphorylates SV40 LT at Ser^111^ and Ser^112^ producing an augment of 50-fold of the rate of nuclear import of its protein [40]. In the Che-1 308–325 aa region, two consecutive CK2 consensus sites (S/T-x-x-D/E/pS) are located in the proximity of its NLS [41, 42]. To investigate whether Che-1 could be a new CK2 target, we started testing if Che-1 physically interacts with CK2. As shown in Fig. 5A and Supplementary Figure 4A, immunoprecipitation experiments revealed that Che-1 co-precipitates with CK2. Next, we analysed the role of CK2 in Che-1 phosphorylation by performing 2D-electrophoresis experiments in HeLa cells treated or not with the CK2 inhibitor, 4,5,6,7-Tetrabromobenzotriazole (TBB). From this experiment, we observed a strong decrease in Che-1 phosphorylation in the absence of CK2 kinase activity (Fig. 5B and Supplementary Figure 4B). Consistent with these findings, the depletion of CK2 expression by using specific siRNA stealth oligonucleotides (siCK2) induced a remarkable reduction of Che-1 phosphorylation when compared to control cells (Fig. 5C and Supplementary Figure 4C). Next, we sought to determine the possibility that Che-1 was a direct substrate of CK2. To this aim, GST-Che-1 fusion protein was incubated or not with recombinant CK2 enzyme and subjected to in vitro kinase assay. The kinase reaction products were analysed by nano-HPLC-MS/MS and the results obtained were then submitted to the SEQUEST search engine against the Uniprot Human protein database using the phosphoRS tool. This approach allowed identifying four Che-1 phosphopeptides, including those containing Ser^316^, Ser^320^ and Ser^321^ (Table 1 and Supplementary Figure 4D). As expected, no phosphopeptides were detected when Che-1 was incubated in the absence of CK2 (data not shown).

Next, we verified the effect of CK2 silencing on Che-1 cellular localization by performing immunofluorescence experiments. As shown in Fig. 5D and Supplementary Figure 4E, the absence of protein kinase does not affect nuclear localization of Che-1. Then, in order to confirm the role of these phosphorylations in Che-1 activity, we tested whether the absence of CK2 could affect Che-1/histone H3 binding. Co-immunoprecipitation experiments in HCT116 cells treated or not with TBB, demonstrated that the inhibition of CK2 kinase activity induces a drastic reduction of histone H3 interaction with Che-1 (Supplementary Figure 4F), and similar results were obtained following CK2 depletion (Fig. 5E and Supplementary Figure 4G).

Altogether, these data clearly demonstrate that CK2 phosphorylates Che-1 at Ser^316^, Ser^320^ and Ser^321^, and that these modifications are essential for Che-1 activity.

## Discussion

Thanks to its ability to interact with several transcription factors, Che-1 represents an important cofactor involved in multiple cellular processes [7, 12, 13]. In this context, post-translational modifications play a crucial role in controlling Che-1 co-transcriptional activity in response to different stimuli [6, 16]. We recently described Che-1 involvement in MM proliferation by affecting chromatin structure and sustaining global gene transcription. Also, we demonstrated that 308-325 aa region of Che-1 is required for these activities [15].

**Fig. 6:**
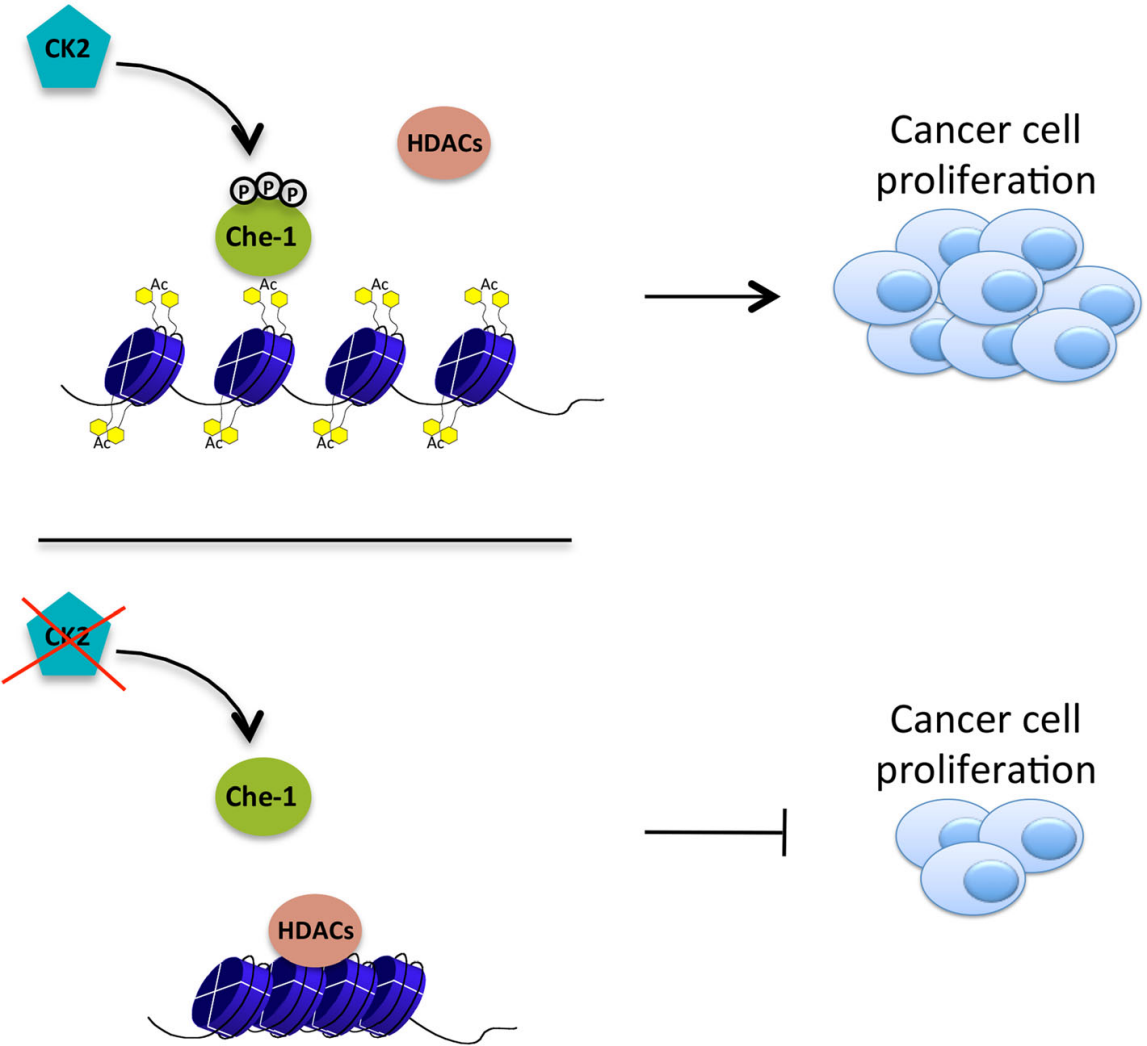
Role of CK2 phosphorylation in regulating Che-1 functions. Phosphorylation of Che-1 by CK2 is required for displacing HDACs from chromatin and sustaining cancer cell proliferation

In this study, we extended these results, confirming the roles played by Che-1 in the control of cellular proliferation and chromatin remodelling, in other cancer cell lines. More importantly, our results highlighted that the phosphorylations at Ser^316^, Ser^320^ and Ser^321^, contained in the Che-1 308-325 aa region, are essential for Che-1 activity. Consistent with these findings, a Che-1 mutant lacking these residues (Che-1 3S) is unable to bind histones and to regulate chromatin structure and transcription. Finally, we identified CK2 as the protein kinase responsible for Che-1 phosphorylation (Fig. 6).

The relevance of 308-325 aa region in controlling Che-1’s functions has already been highlighted [12, 15]. This region is highly conserved among several species and is present in many proteins involved in transcriptional regulation [12, 14]. In addition, phosphoproteomic studies have demonstrated Ser^316^, Ser^320^ and Ser^321^ phosphorylation in all species analysed and that this modification is strongly abolished by the inhibition of different protein kinases implicated in cell cycle control [36–38]. In this study, we demonstrate the functional relevance of these modifications, since the replacement of these three serines produces a protein unable to modulate chromatin structure and gene transcription. The experiments conducted with the nuclease micrococcus demonstrate that this mutant has a dominant-negative behaviour compared to control, nevertheless we have not found the same effect on transcription and cell growth. This observation may be explained by the ability of cells to implement alternative strategies capable of supporting the effects of 3S mutant. Otherwise, it could be possible that the effects of Che-1 on cell proliferation are also the result of other mechanisms. Our data show that Che-1 mutant does not bind histone proteins, and presumably serine phosphorylation is necessary to increase the acidic components of this region, thus enhancing the binding with the basic structure of histones. The importance of this region and its modifications is further confirmed by the analysis of the SV40 Large T protein (SV40 LT). This oncoprotein contains a region of high homology with the 308-325 aa region of Che-1, and also it is able to bind histones, sustaining their acetylation, and to activate cell proliferation by regulating gene transcription [39]. By producing a SV40 LT mutant protein, we were able to demonstrate that even in this case, the phosphorylation of these serines is necessary for the functionality of the protein, thus supporting the hypothesis of a close correlation between viral oncoproteins and Che-1. Finally, we demonstrated that Che-1 is phosphorylated by CK2 at Ser^316^, Ser^320^ and Ser^321^ in proliferating cells although in which phase of the cycle these modifications occurs, remain to be clarified. CK2 is highly expressed in several tumor cells, and its activity sustains cell proliferation [21–24], therefore it is considered an attractive target to counteract drug resistance in cancer. Indeed, specific CK2 inhibitors have shown proven efficacy on tumor cell proliferation and drug resistance [19]. Since Che-1 silencing is able to induce apoptosis or cell cycle arrest and sensitize MM cells to treatment with proteasome and BET inhibitors [2, 15], targeting Che-1 by using CK2 inhibitors could represent an interesting novel therapeutic approach.

## Conclusion

Taken together, our data provide evidence about the phosphorylation mechanisms underlying the biological role of Che-1. Our results confirm that Che-1 displays a pro-proliferative effect by inducing global histone acetylation not only in MM cells. Specifically, we point out the relevance of Che-1 region 308-325 for this function, by producing a defective mutant lacking of three serine residues (316, 320 and 321). Moreover, based on homology sequence with SV40 LT, we identified CK2 as the protein kinase responsible for Che-1 phosphorylation at Ser^316^, Ser^320^ and Ser^321^. The results herein obtained suggest a novel strategy for cancer treatment focused on the inhibition of Che-1-CK2 axis.

## Supplementary Information

**Additional file 1: Supplementary Figure 1.** Che-1 depletion reduces cell proliferation. **A**: WB analysis with the indicated antibodies of total cell extracts from HeLa cells transiently transfected with siControl or siChe-1 for indicated times. **B**: Annexin V-FITC apoptosis assay in HeLa cells transfected with siControl and siChe-1 for 48 h and then subjected to flow cytometry (left). Representative WB showing the transfection efficiency of Che-1 silencing (right). **C**: Bar plot showing the differences in cell viability of the cell lines shown in Fig. 1A. **D**: Replicate blots relative to WB shown in Fig. 1E and used for densitometry. **E**: Cell number-normalized quantification of total RNA extracted from the indicated cell lines used in Fig. 1A. Statistical significance is indicated by asterisks as follow: *P < 0.05, **P < 0.01, ***P < 0.005, ****P < 0.001, n.s. = not significant.

**Additional file 2: Supplementary Figure 2.** Che-1 is highly phosphorylated. **A**: Two different replicates of 2D- experiments of total cell extracts from HeLa cells treated or not with λ-PP related to Fig. 2B. **B**: Two different replicates of 2D-Gel electrophoresis (top) and representative WB (bottom) of HeLa cells transiently transfected with Myc-Che-1 or Myc-Che-1 3S, related to Fig. 2D.

**Additional file 3: Supplementary Figure 3.** Che-1 phosphorylation is required for its pro-proliferative ability. **A**: WB analysis with the indicated antibodies showing the transfection efficiency of the experiment described in Fig. 3A. **B**, **C** and **D**: Cell proliferation analysis (left) and relative WB analysis (right) of KMS27, HeLa and 293 T cells transiently transfected with Myc-Che-1, Myc-Che-1 3S or control vector (pCS2MT). Bar plot shows the average number of cells observed in these experiments (*n* = 5). **E**: Cell number-normalized total RNA quantification of the indicated cell line transiently transfected with Myc-Che-1 wt, 3S mutant or control vector. Error bars represent the SD of triplicate experiments (*n* = 3). **F**: Nuclear extracts from HCT116 cells transiently transfected with Myc-Che-1 wt or 3S and subjected to IP with H3 antibody. Immunoprecipitated complexes were then analysed by WB with the indicated antibodies. Input corresponds to 10% of the nuclear extracts used for IP. **G**: Cell proliferation analysis of KMS27 cells transiently transfected with the indicated peptides. Bar plot shows the average number of cells observed in these experiments (*n* = 3). Statistical significance is indicated by asterisks as follow: *P < 0.05, **P < 0.01, ***P < 0.005, ****P < 0.001, n.s. = not significant.

**Additional file 4: Supplementary Figure 4. **CK2 phosphorylates Che-1. **A**: Nuclear extracts from HCT116 cells were subjected to IP with CK2 antibody. Immunoprecipitated complexes were then analysed by WB with the indicated antibodies. Input corresponds to 10% of the nuclear extracts used for IP. **B** and **C**: Representative WB analyses of total cell extracts showing the transfection efficiency of the experiments described in Fig. 5B and C. **D**: The panel shows the MS/MS spectrum of the phosphopeptide YLVDGTKPNAGSEEISSEDDELVEEK identifying the phosphorylation of Che-1 residues S320 and S321. **E**: Representative WB analyses of total cell extracts showing the transfection efficiency of the experiment shown in Fig. 5D. **F**: Nuclear extracts from HCT116 cells treated or not with 80 μM TBB for 4 h and then subjected to IP with Che-1 antibody. Immunoprecipitated complexes were then analysed by WB with the indicated antibodies. Input corresponds to 10% of the nuclear extracts used for IP. **G**: WB of the IP experiments used for densitometryc analysis shown in Fig. 5E.

**Additional file 5: Supplementary Table 1.** Complete list of antibodies used in this study.

## Data Availability

The data that support this study are available from the corresponding author upon reasonable request.

## Abbreviations

MM: Multiple myeloma
HCC: Hepatocellular carcinoma
CK2: Casein Kinase II
SV40 LT: Simian virus 40 Large T
Rb: Retinoblastoma
WB: Western blot
IP: Immunoprecipitation
MNase: Micrococcal nuclease
MS: Mass Spectrometry
ATP: Adenosine 5′-triphosphate
λ-PP: Lambda protein phosphate
wt: Wild type
Ser: Serine
aa: Aminoacids
NLS: Nuclear localization signal
PI: Propidium iodide
5-FUrd: 5-fluorouridine
