# Supplementary figures and images for: CK2-mediated phosphorylation of Che-1/AATF is required for its pro-proliferative activity
Source: J Exp Clin Cancer Res. 2021 Jul 15;40:232. doi: 10.1186/s13046-021-02038-x (PMC8281565; doi:10.1186/s13046-021-02038-x)

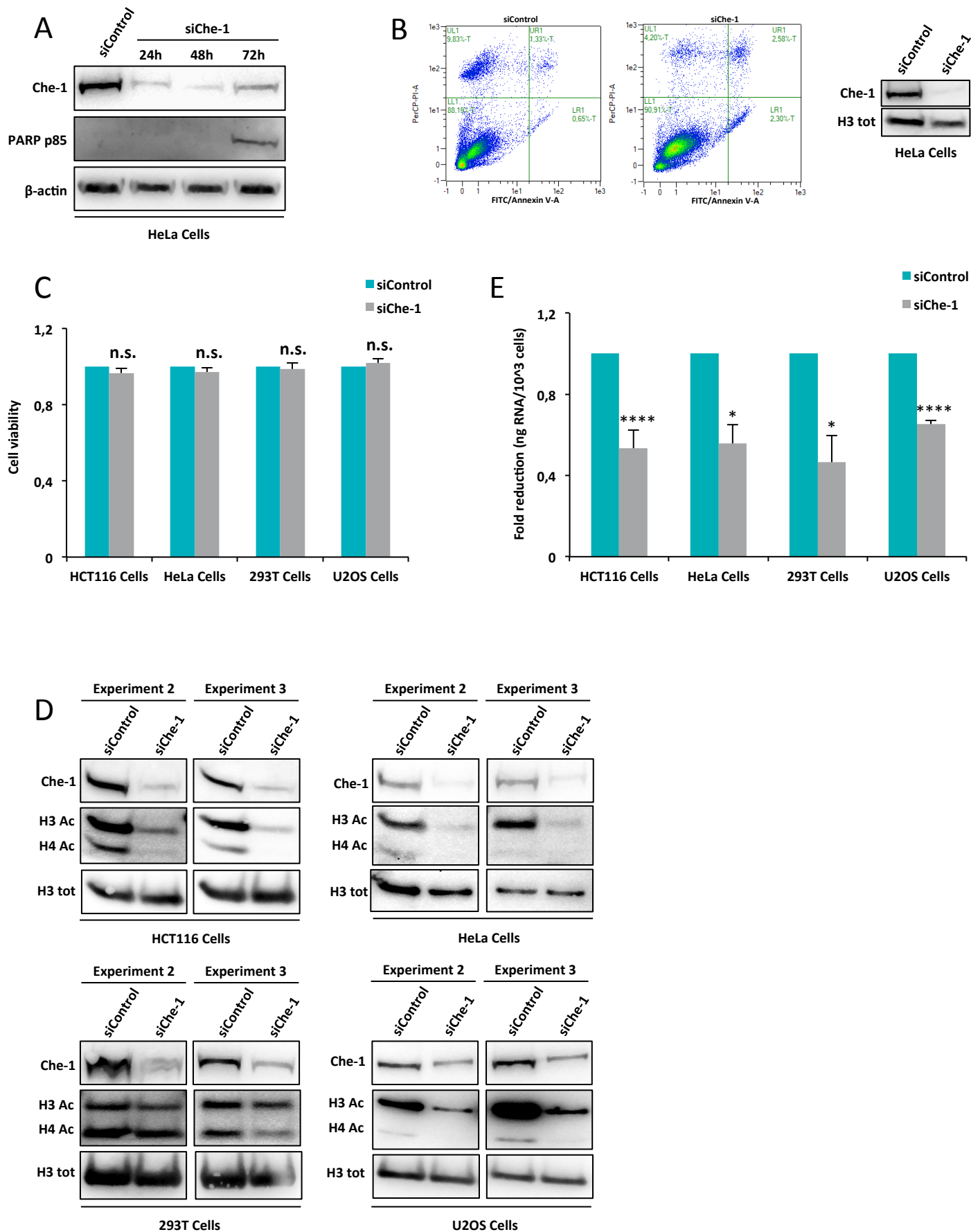

Supplementary Figure 1

Supplement: Supplementary file 1 — Additional file 1: Supplementary Figure 1. Che-1 depletion reduces cell proliferation. A: WB analysis with the indicated antibodies of total cell extracts from HeLa cells transiently transfected with siControl or siChe-1 for indicated times. B: Annexin V-FITC apoptosis assay in HeLa cells transfected with siControl and siChe-1 for 48 h and then subjected to flow cytometry (left). Representative WB showing the transfection efficiency of Che-1 silencing (right). C: Bar plot showing the differences in cell viability of the cell lines shown in Fig. 1A. D: Replicate blots relative to WB shown in Fig. 1E and used for densitometry. E: Cell number-normalized quantification of total RNA extracted from the indicated cell lines used in Fig. 1A. Statistical significance is indicated by asterisks as follow: *P < 0.05, **P < 0.01, ***P < 0.005, ****P < 0.001, n.s. = not significant. [file 13046_2021_2038_MOESM1_ESM.pdf]

A

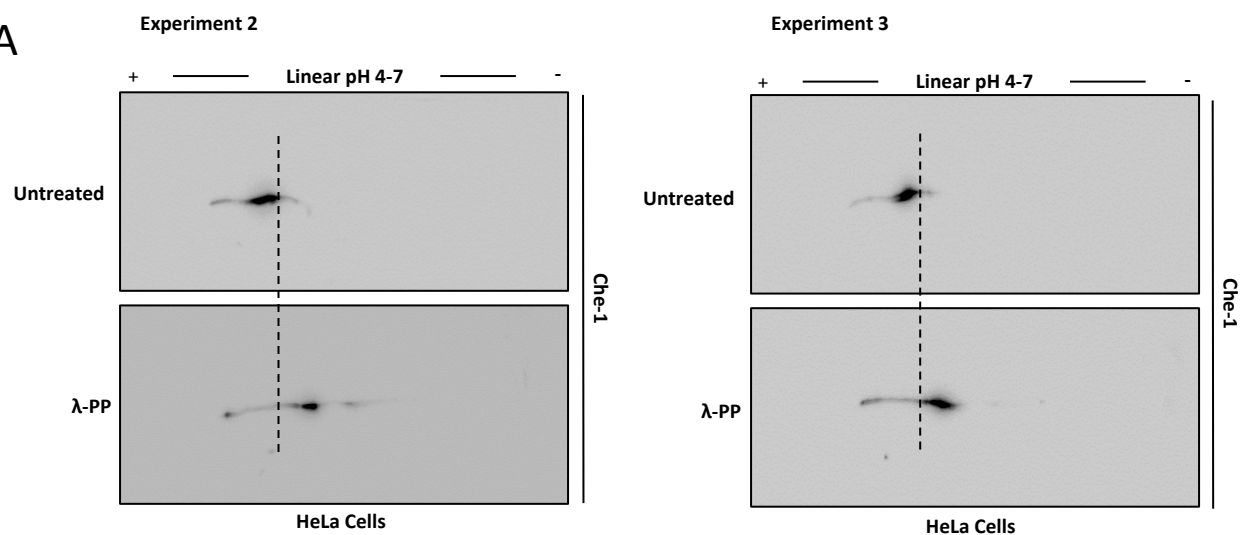

B

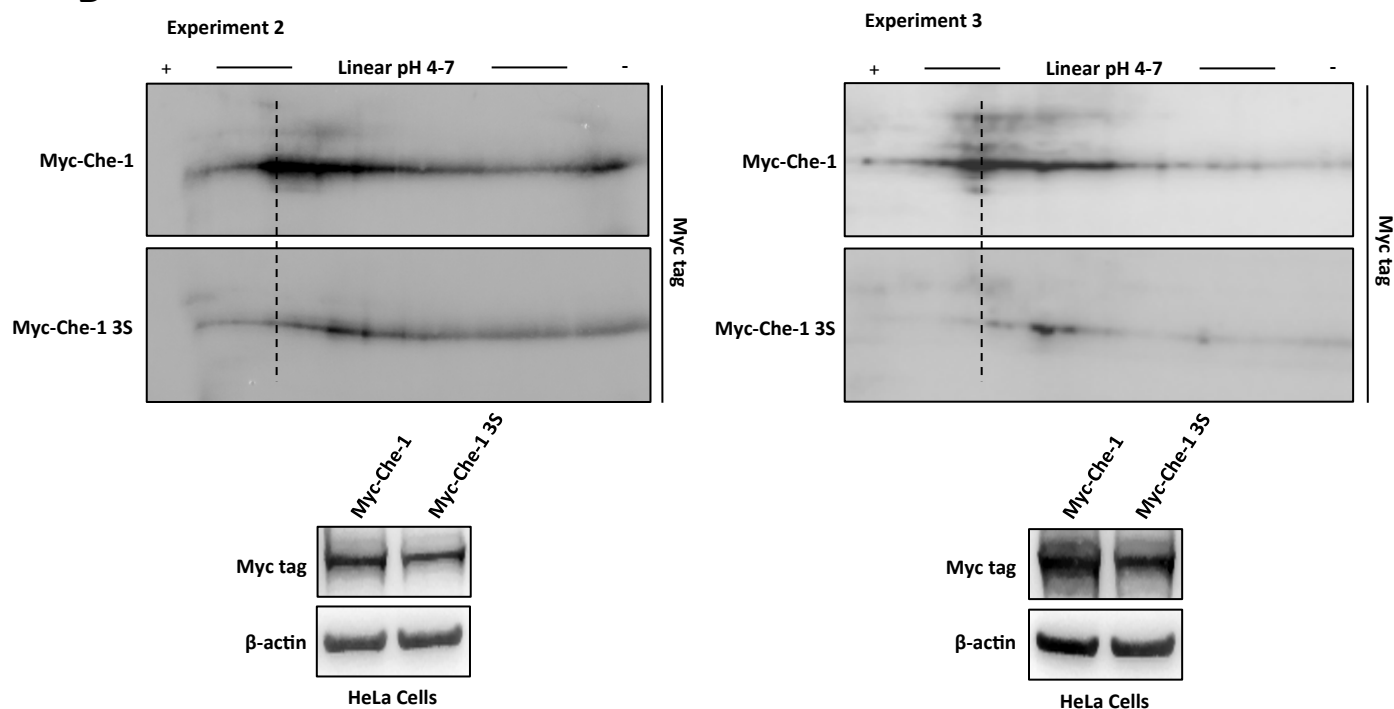

Supplement: Supplementary file 2 — Additional file 2: Supplementary Figure 2. Che-1 is highly phosphorylated. A: Two different replicates of 2D- experiments of total cell extracts from HeLa cells treated or not with λ-PP related to Fig. 2B. B: Two different replicates of 2D-Gel electrophoresis (top) and representative WB (bottom) of HeLa cells transiently transfected with Myc-Che-1 or Myc-Che-1 3S, related to Fig. 2D. [file 13046_2021_2038_MOESM2_ESM.pdf]

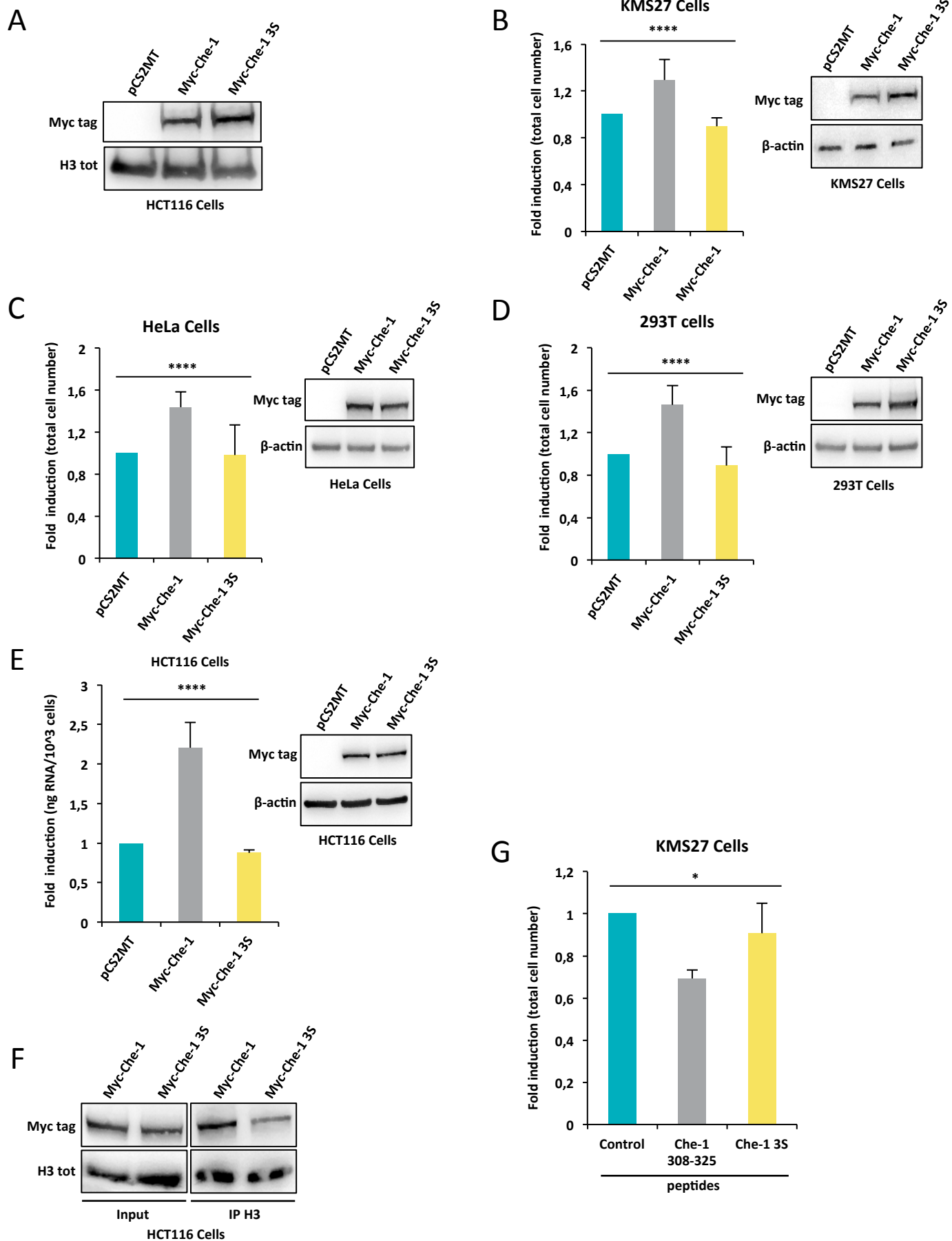

Supplementary Figure 3

Supplement: Supplementary file 3 — Additional file 3: Supplementary Figure 3. Che-1 phosphorylation is required for its pro-proliferative ability. A: WB analysis with the indicated antibodies showing the transfection efficiency of the experiment described in Fig. 3A. B, C and D: Cell proliferation analysis (left) and relative WB analysis (right) of KMS27, HeLa and 293 T cells transiently transfected with Myc-Che-1, Myc-Che-1 3S or control vector (pCS2MT). Bar plot shows the average number of cells observed in these experiments (n = 5). E: Cell number-normalized total RNA quantification of the indicated cell line transiently transfected with Myc-Che-1 wt, 3S mutant or control vector. Error bars represent the SD of triplicate experiments (n = 3). F: Nuclear extracts from HCT116 cells transiently transfected with Myc-Che-1 wt or 3S and subjected to IP with H3 antibody. Immunoprecipitated complexes were then analysed by WB with the indicated antibodies. Input corresponds to 10% of the nuclear extracts used for IP. G: Cell proliferation analysis of KMS27 cells transiently transfected with the indicated peptides. Bar plot shows the average number of cells observed in these experiments (n = 3). Statistical significance is indicated by asterisks as follow: *P < 0.05, **P < 0.01, ***P < 0.005, ****P < 0.001, n.s. = not significant. [file 13046_2021_2038_MOESM3_ESM.pdf]

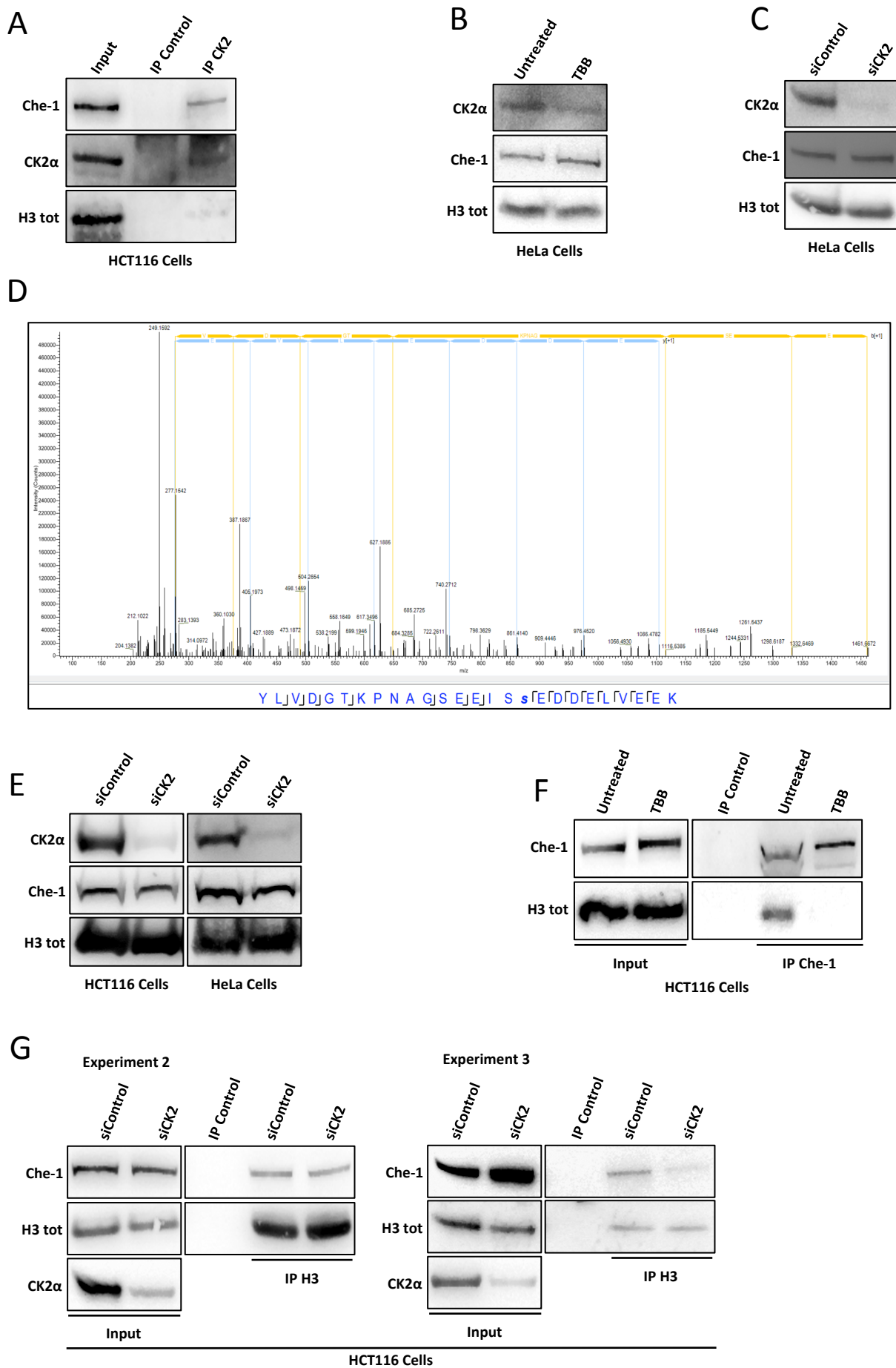

Supplement: Supplementary file 4 — Additional file 4: Supplementary Figure 4. CK2 phosphorylates Che-1. A: Nuclear extracts from HCT116 cells were subjected to IP with CK2 antibody. Immunoprecipitated complexes were then analysed by WB with the indicated antibodies. Input corresponds to 10% of the nuclear extracts used for IP. B and C: Representative WB analyses of total cell extracts showing the transfection efficiency of the experiments described in Fig. 5B and C. D: The panel shows the MS/MS spectrum of the phosphopeptide YLVDGTKPNAGSEEISSEDDELVEEK identifying the phosphorylation of Che-1 residues S320 and S321. E: Representative WB analyses of total cell extracts showing the transfection efficiency of the experiment shown in Fig. 5D. F: Nuclear extracts from HCT116 cells treated or not with 80 μM TBB for 4 h and then subjected to IP with Che-1 antibody. Immunoprecipitated complexes were then analysed by WB with the indicated antibodies. Input corresponds to 10% of the nuclear extracts used for IP. G: WB of the IP experiments used for densitometryc analysis shown in Fig. 5E. [file 13046_2021_2038_MOESM4_ESM.pdf]
