# Supplementary material for: CK2-mediated phosphorylation of Che-1/AATF is required for its pro-proliferative activity
Source: J Exp Clin Cancer Res. 2021 Jul 15;40:232. doi: 10.1186/s13046-021-02038-x (PMC8281565; doi:10.1186/s13046-021-02038-x)
Supplement: Supplementary file 5 — Additional file 5: Supplementary Table 1. Complete list of antibodies used in this study. [file 13046_2021_2038_MOESM5_ESM.docx]

| Rabbit Polyclonal Antibodies | | |
| --- | --- | --- |
|  | **Source** | **Cat. Number** |
| Che-1 | Fanciulli et al., 2000 |  |
| Che-1 | Bethyl | A301-032A |
| Histone H3 | Abcam | ab18521 |
| Acetyl Histone H4 | Millipore | 06-866 |
| Acetyl Histone H3 (1-20) | Millipore | 382158 |
| Histone H3K9me3 | Abcam | ab8898 |
| Cyclin B1 | Sigma | C8831 |
| CK2α | Cell Signaling | 2656 |
| SV40 LT (D1E9E) | Cell Signaling | 15729 |
|  |  |  |
| Mouse Monoclonal Antibodies | | |
|  | **Source** | **Cat. Number** |
| α - tubulin | Calbiochem | CP06 |
| β - actin (AC-15) | Sigma-Aldrich | A5441 |
| Myc 9e10 | Thermo Fisher Scientific | MA1-980 |
| BrdU | Sigma-Aldrich | B8434 |
| Cleaved PARP (Asp214) (TC9) | Cell Signaling | 9548 |
|  |  |  |

**Supplementary Table 1.** Complete list of antibodies used in this study
